# Capitalizing on the Autophagic Response for Treatment of Liver Disease Caused by Alpha-1-Antitrypsin Deficiency and Other Genetic Diseases

**DOI:** 10.1155/2014/459823

**Published:** 2014-06-03

**Authors:** Andrew S. Chu, David H. Perlmutter, Yan Wang

**Affiliations:** ^1^Department of Pediatrics, University of Pittsburgh School of Medicine, Pittsburgh, PA 15224, USA; ^2^Children's Hospital of Pittsburgh of UPMC, Pittsburgh, PA 15224, USA; ^3^Department of Cell Biology, University of Pittsburgh School of Medicine, Pittsburgh, PA 15261, USA

## Abstract

Alpha-1-antitrypsin deficiency (ATD) is one of the most common genetic causes of liver disease and is a prototype of liver diseases caused by the pathologic accumulation of aggregated mutant alpha-1-antitrypsin Z (ATZ) within liver cells. In the case of ATD-associated liver disease, the resulting “gain-of-function” toxicity can lead to serious clinical manifestations, including cirrhosis and hepatocellular carcinoma. Currently, the only definitive therapy for ATD-associated liver disease is liver transplantation, but recent efforts have demonstrated the exciting potential for novel therapies that target disposal of the mutant protein aggregates by harnessing a cellular homeostasis mechanism called autophagy. In this review, we will summarize research advances on autophagy and genetic liver diseases. We will discuss autophagy enhancer strategies for liver disease due to ATD and another genetic liver disease, inherited hypofibrinogenemia, caused by the proteotoxic effects of a misfolded protein. On the basis of recent evidence that autophagy plays a role in cellular lipid degradation, we also speculate about autophagy enhancer strategies for treatment of hepatic lipid storage diseases such as cholesterol ester storage disease.

## 1. Introduction

Alpha-1-antitrypsin deficiency (ATD) is one of the most common genetic causes of liver disease. It is characterized by accumulation of a misfolded secretory protein in the endoplasmic reticulum of liver cells. In some affected homozygotes this “proteotoxic” state leads to hepatic fibrosis/cirrhosis and hepatocellular carcinoma. Investigations in a variety of model systems have shown that macroautophagy is activated when the mutant *α*1-antitrypsin molecule, *α*1-antitrypsin Z (ATZ), accumulates in cells and autophagy plays a key role in intracellular degradation of ATZ. Therapeutic strategies that enhance autophagy, using either drugs or gene transfer with a transcriptional activator of autophagy, have recently been shown to reverse hepatic accumulation of misfolded protein and hepatic fibrosis in a mouse model of ATD. In this review we will discuss autophagy enhancer strategies for liver disease due to ATD and another genetic liver disease, inherited hypofibrinogenemia, caused by the proteotoxic effects of a misfolded protein. On the basis of recent evidence that autophagy plays a role in cellular lipid degradation, we also speculate about autophagy enhancer strategies for treatment of hepatic lipid storage diseases such as cholesterol ester storage disease.

## 2. Mechanisms of Liver Disease in ATD

*α*1-Antitrypsin (AT) is an abundant serum glycoprotein predominantly synthesized by liver parenchymal cells. It is a prototype member of the serine protease inhibitor family, known as serpins, with a particular strong profile for inhibiting neutrophil elastase, cathepsin G, and proteinase 3. The classical form of ATD (referred to as PIZZ) results from a point mutation in the AT gene that leads to a lysine to glutamate substitution at residue 342 of the protein that renders the mutant AT protein (termed ATZ) prone to misfolding. A number of studies have shown that ATZ is also prone to polymerization and aggregation, and it is likely that this aggregation-prone tendency plays a distinct role in the nature of the pathology of the disease [1]. The characteristic histologic finding in the liver is periodic acid-Schiff- (PAS-) positive, diastase-resistant globules in hepatocytes [2], representing the accumulation of ATZ within the early compartments of the secretory pathway. Because of misfolding, the mutant form of AT is inefficient in traversing the secretory pathway, and so there is a substantial reduction of AT in the systemic circulation, such that serum AT levels are approximately 10–15% of normal. Over the years, studies of this deficiency have led to the conclusion that the two consequences of misfolding are responsible for the two major clinical manifestations. Decreased serum levels of AT are primarily responsible for lung damage because there is loss of antineutrophil elastase function and neutrophil elastase can destroy the extracellular matrix of the lung. Intracellular accumulation of ATZ in the liver is primarily responsible for liver damage by gain-of-function proteotoxicity. The gain-of-function mechanism for liver disease is best demonstrated by transgenic mouse models [3, 4] that strongly recapitulate many of the features of the human liver disease even though they have normal levels of endogenous murine AT, eliminating the possibility that loss of AT function contributes to liver damage [5, 6].

The clinical presentation of ATD-associated liver disease is highly variable and may become apparent in distinct forms during infancy, childhood years, adolescence, or at 50–65 years of age [1]. Patients who exhibit features of liver disease during infancy can present within the first two months of life with persistent jaundice, hepatomegaly, and elevated serum conjugated bilirubin and transaminase levels. A prospective Swedish national screening study initiated in the 1970s provides important insights into the natural history of ATD [7]. In that study, Sveger et al. screened 200,000 newborns, of whom 120 were ZZ homozygotes. A follow-up study of a subset of 26-year-old individuals from this cohort determined that less than ten percent of the cohort had experienced clinically significant liver disease [8]. A limitation of this cohort study is the lack of liver biopsy data, which makes it difficult to assess whether asymptomatic homozygotes can have subclinical pathology for decades that eventually presents as “adult” ATD-associated liver disease. However, the study clearly demonstrated that only a subgroup of affected homozygotes are susceptible to clinically significant liver disease in the first three decades of life and led to the recognition of the importance of putative genetic and environmental modifiers in liver disease pathogenesis.

In adults, ATD-associated liver disease may be recognized either because of portal hypertension or because of the detection of hepatocellular carcinoma (HCC). HCC is an important manifestation that is likely caused by chronic proteotoxicity that drives liver fibrosis and hyperproliferation of hepatocytes. Support for this hypothesis comes from experimental studies that have found that ATZ accumulation in hepatocytes is associated with impaired cell proliferation and chronic regenerative signaling [9]. A Swedish autopsy study conducted by Eriksson et al. revealed a highly significant association of ATD with HCC beyond what would be expected with cirrhosis alone [10]. In the past two decades, there has been increased recognition of “adult” ATD-associated liver disease. Over the last ten years, 85 to 90 percent of liver transplants for ATD performed in the US were for adults, most commonly around 50 to 65 years of age (United Network of Organ Sharing, personal communication).

## 3. Role of Autophagy in ATD Liver Disease

To understand the mechanism of the liver disease associated with ATD, our laboratory focused initially on mechanisms by which mutant ATZ is degraded when it accumulates within cells. Early studies using yeast and mammalian cell lines showed that the proteasomal pathway participated in intracellular degradation of mutant ATZ [11–13]. Indeed mutant ATZ was one of the first identified substrates of the pathway that is now known as ER-associated degradation (ERAD). Nevertheless, there was evidence from these studies that the proteasomal pathway could not completely account for the disposal of ATZ. Studies in human cell line models of ATD first led to the recognition that autophagy was activated by intracellular accumulation of ATZ, and later it was shown that autophagy participated as a second pathway for disposal of mutant ATZ [14, 15]. Autophagy refers to distinct intracellular catabolic pathways that mediate degradation of unnecessary or dysfunctional cellular components through the machinery of lysosomes. These pathways act, at least in part, as protective and survival mechanisms under the condition of nutrient starvation or other stress states. Autophagy also appears to play critical roles in homeostasis, cell growth, and differentiation. According to the mechanism by which the substrates are delivered to the lysosomes, autophagy can be divided into three subtypes: macroautophagy, microautophagy, and chaperone-mediated autophagy. The studies investigating autophagy in ATD have focused exclusively on macroautophagy. Macroautophagy is characterized by the formation of double membrane vacuoles in the cytoplasm, also called autophagosomes, which then fuse with lysosomes for degradation of the internal constituents and generation/recycling of new amino acids for cell survival.

**Figure 1:**
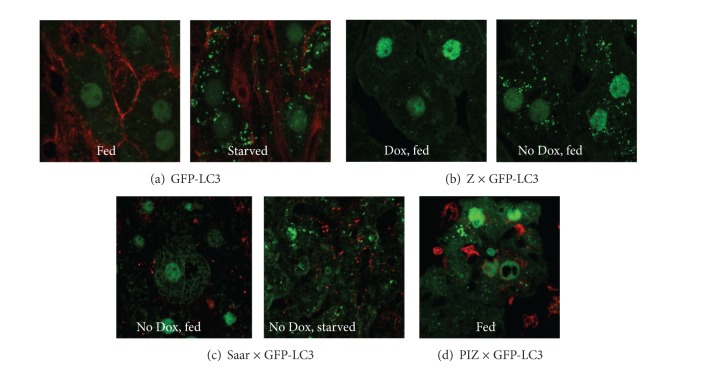
Accumulation of ATZ in liver specifically activates autophagy. Liver sections are stained with anti-GFP to enhance the fluorescent signal. (a) Sections from the GFP-LC3 mouse have GFP+ autophagosomes only in the starved state (right panel—starved for 24 h). (b) Sections from the Z × GFP-LC3 mouse have GFP+ autophagosomes; however, the mouse is fed, but only after doxycycline (Dox) is removed from the drinking water so that the ATZ gene is expressed (right panel). (c) Sections from the Saar × GFP-LC3 mouse (lower right) do not show autophagosomes when the AT Saar variant is expressed following withdrawal of Dox in the fed state, but starvation does lead to GFP+ autophagosomes (right panel). (d) GFP+ autophagosomes are present in the PiZ × GFP-LC3 mouse in the fed state, as this mouse has constitutive expression of ATZ. Reprint from reference [1] with permission.

Autophagy was firstly implicated in ATD when increased autophagosomes were observed in human fibroblast cell lines engineered for expression of ATZ [14]. Increased autophagic vacuoles were also observed in the liver of PiZ transgenic mice and in liver biopsy specimens from patients with ATD [14]. This led to the conclusion that autophagy was activated by intracellular accumulation of ATZ in model systems and in affected tissues from the disease and also raised the possibility that autophagy could participate in degrading mutant ATZ. In initial studies, we showed that degradation of ATZ in human cell line models was partially abrogated by chemical inhibitors of autophagy, such as 3-methyladenine, wortmannin, and LY-294002 [14]. Later we provided definitive genetic evidence using an embryonic fibroblast cell line (MEF) from an ATG5-null mouse engineered for expression of ATZ [15]. The results from this study showed that the degradation rate of ATZ was attenuated in ATG5-null cells compared to the wild-type MEFs. Furthermore, we observed massive accumulation of ATZ with very large inclusions in the ATG5-null cell line. Thus, in addition to providing definitive evidence that autophagy participates in the degradation of ATZ, these data suggest that it also plays a homeostatic role in ATD state by preventing the toxic cytoplasmic accumulation of ATZ through piecemeal digestion of insoluble aggregates [15]. Further, by breeding a mouse model with liver-specific inducible expression of ATZ onto the GFP-LC3 mouse background that is characterized by GFP+ autophagosomes, we found that accumulation of ATZ in liver cells is sufficient to activate autophagy [15]. The effect of ATZ accumulation that leads to activation of autophagy is also specific and probably reflects the aggregation-prone properties of ATZ because accumulation of the nonpolymerogenic variant, AT Saar, does not activate autophagy (Figure 1).

Another series of studies using a completely different system for modeling ATD, yeast expressing human ATZ, also showed the importance of autophagy [16]. These studies showed marked delay in degradation of human ATZ in autophagy-deficient yeast strains. Interestingly, the delay in ATZ degradation was most apparent when ATZ was expressed at high levels [16]. These results suggested that when ATZ is predominantly soluble at lower levels of expression, it could be degraded by proteasome, whereas, at higher levels of expression, it is more likely to be associated with chronic accumulation of insoluble polymers or aggregates, in which case autophagy is needed. Recent studies have also demonstrated the role of autophagy in a novel* C. elegans* model of ATD [17].

In addition to the proteasomal and autophagic pathways for disposal of ATZ, a recent study showed that a pathway from Golgi complex to lysosome that involves the endosomal protein sorting receptor sortilin plays a role in degrading intracellular ATZ [18]. We suspect that there are still other mechanisms by which cells degrade mutant ATZ that have not yet been identified.

To understand the molecular mechanism by which the cells mitigate the proteotoxicity of intracellular ATZ accumulation and presumably protect ATD hosts from liver disease, we also focused on the signaling pathways that modulate proteostasis. Using mammalian cell line and mouse models with inducible expression of ATZ and genomic analysis, we found that the NF-*κ*B and TGF-*β* signaling pathways are part of the distinct gene expression profile associated with intracellular accumulation of ATZ, and these pathways are likely central to the hyperproliferation and fibrosis, respectively, which characterizes the hepatic pathology of ATD [19, 20]. Genomic analysis also demonstrated upregulation of regulator of G signaling 16 (RGS16), and we have subsequently found that RGS16 is a specific marker for the proteotoxic state created by intracellular accumulation of ATZ and may be one of the mediators by which autophagy is activated in the liver [21].

## 4. New Autophagy Enhancer Strategies for Treatment of ATD Liver Disease

Because autophagy is activated when mutant ATZ accumulates in cells and participates in intracellular disposal of ATZ, we reasoned that it was a potentially attractive target for drug therapy to mitigate the proteotoxicity that causes liver damage in ATD. From a list of drugs that have been purported to have autophagy enhancer properties, we investigated the effect of carbamazepine (CBZ) in cell line and mouse models of ATD. We selected CBZ because it is approved by the US Food and Drug Administration (FDA) with an extensive safety profile from its use as anticonvulsant and mood stabilizer. We found that CBZ enhanced autophagy and mediated a marked increase on the degradation of mutant ATZ in cell line models [6]. More importantly, CBZ mediated a reduction on hepatic ATZ load and ameliorated hepatic fibrosis in the PiZ mouse model of ATD [6]. These results validated the concept of using autophagy enhancer drugs that target an endogenous presumably protective proteostasis mechanism as a therapeutic strategy. Furthermore, because it is already FDA-approved, this drug could be moved immediately into a phase II/III trial for treatment of severe liver disease due to ATD. Although the lowest effective dose of CBZ (200 mg/kg/day) in mice was considerably higher than the doses used in humans (10–20 mg/kg/day) effective doses of drugs can be 10 to 20 times as high in mice because of the higher ratio of surface area to body weight when compared to humans. The current Phase II/III trial for use of CBZ in severe liver disease due to ATD already ongoing uses doses of CBZ commonly used in clinical medicine, 1200 mg/day for subjects over 15 years of age.

The concept that autophagy enhancer drugs counteract the accumulation and proteotoxicity of misfolded proteins has been further substantiated by the results from an automated high-content screening using a novel* C. elegans* model of ATD [17]. The initial screen of 1280 compounds in the Library of Pharmacologically Active Compounds (LOPAC) identified 5 hit compounds that mediate dose-dependent reduction of ATZ load in this worm model. Interestingly, four of these five hits showed the property of enhancing autophagy. Another interesting observation from these results is that two of these compounds (fluphenazine and pimozide) are from the phenothiazine family, which is structurally related to the tricyclic antidepressants family of which CBZ is a member. We have further validated the effect of fluphenazine (Flu) in mammalian cellular and mouse models of ATD [22]. In nanomolar concentrations, Flu enhanced the degradation of ATZ and reduced the cellular ATZ load in human cellular models of ATD. In PiZ mouse model, Flu reduced the proteotoxicity of ATZ accumulation* in vivo* and mediated a decrease in hepatic fibrosis [22]. As Flu is a FDA-approved drug that is in active clinical use, like CBZ, it could be immediately tested in clinical trials, “repurposing” for treating ATD-associated liver disease. In addition, several other autophagy enhancer drugs, including overlapping hits, have been identified from two independent high-throughput screenings [23, 24]. The identified compounds from these screenings showed enhanced autophagic degradation of aggregation-prone protein* huntingtin* in different models of Huntington's disease. Furthermore, several compounds identified in these screenings are in the phenothiazine family, for example, fluphenazine, pimozide, and fluspirilene [17, 23, 24]. Thus the results from these screenings provide further evidence for the potential therapeutic application of drugs that target endogenous proteostasis mechanisms and identify two new strategies for chemical- and computation-based drug discovery using the autophagy enhancer drug paradigm and the phenothiazine structure.

**Figure 2:**
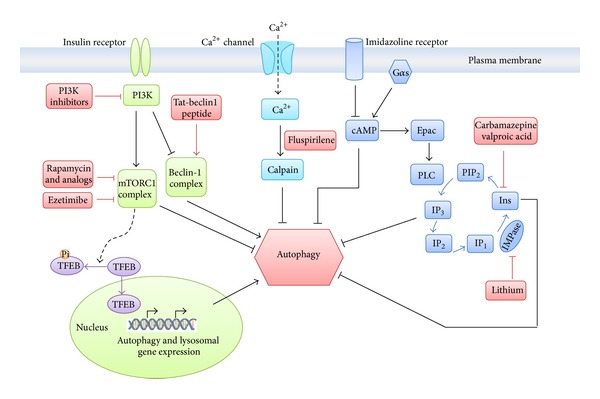
Regulation of autophagy and possible targets of the autophagy enhancer drugs. Drugs that modulate autophagy can be divided into two categories depending on whether or not they act through mTOR, a master negative regulator of autophagy that functions through the formation of mTORC1 complex. This complex is suppressed by specific inhibitors such as rapamycin and its analogs, which therefore enhance autophagy [43]. A recent study showed that ezetimibe perturbed the cholesterol homeostasis and decreased mTOR recruitment to late endosome/lysosome; thus ezetimibe is thought to induce autophagy by suppressing mTORC1 [28]. The mTOR signaling pathway can also be regulated by specific inhibitors target to the upstream of mTOR, for example, PI3K inhibitor [44]. In addition, nuclear translocation of TFEB promoted autophagic degradation of ATZ in PiZ mouse model [33]. There is evidence showing that TFEB interacts with mTORC1 on the lysosomal membrane; because it promotes phosphorylation of TFEB, mTORC1 is now considered an antagonist of TFEB activity [32]. Tat-beclin 1 peptide is identified as a potent inducer of autophagy and enhances the degradation of mutant huntingtin and several invasive bacterial and viral pathogens [30]. Several autophagy enhancer drugs identified from the recent drug screenings, including the phenothiazines, are thought to act on autophagy by mTOR-independent mechanism(s) [17, 23, 24]. One of the phenothiazines that have been investigated, fluspirilene, is thought to induce autophagy by reducing intracellular Ca^2+^ and preventing calpain-1-mediated cleavage of autophagy gene ATG5 [26]. The mood-stabilizing effects of lithium are thought to involve inhibition of IMPase and prevention of inositol recycling, while CBZ and valproic acid appear to act on Ins [24, 25]. The inhibition of IMPase or Ins leads to reduced intracellular inositol and IP3 levels, which therefore induce autophagy. However, the precise mechanism by which autophagy is regulated by the calcium-related signaling pathway or the phosphatidylinositol signaling pathway has not been elucidated. In addition, further work on whether these targets are truly independent of mTOR is needed.

The specific molecular target of CBZ and the phenothiazine drugs that leads to enhanced autophagy of ATZ remains largely unknown. Because the classical mTOR antagonist rapamycin had minimal effects on ATZ disposal in cell line models and did not alter hepatic ATZ levels in the PiZ mouse model in our studies [6], we have surmised that the effects of CBZ on autophagic degradation of ATZ act through an mTOR-independent mechanism. Previous work on the mood- stabilizing effects of CBZ suggests that it is similar to lithium and valproic acid, and all three compounds appear to have autophagy enhancer properties [6, 24, 25]. The mood-stabilizing effects of lithium are thought to involve inhibition of inositol monophosphatase (IMPase) and prevention of inositol recycling, while CBZ and valproic acid appear to act on inositol (1,4,5)-trisphosphate (Ins) [24, 25]. The inhibition of IMPase or Ins leads to reduced intracellular inositol levels and inositol-1,4,5-trisphosphate (IP3) levels, which negatively regulate autophagy (Figure 2). The phenothiazines are also thought to act on autophagy by mTOR-independent mechanism(s). One of the phenothiazines that have been investigated, fluspirilene, is thought to induce autophagy by reducing intracellular Ca^2+^ and preventing calpain-1-mediated cleavage of autophagy gene ATG5 [26]. Nevertheless, further work on the molecular targets of these drugs that mediate activation of autophagy and whether these targets are truly independent of TOR is needed.

Several other drugs have recently been shown to induce autophagy. Glucosamine and N-acetylglucosamine increased autophagy in mammalian cell lines [27]. Although the mechanism of this effect was not elucidated, it was independent of mTOR pathway. Another drug that has recently been shown to activate autophagy specifically is the cholesterol-lowering agent ezetimibe [28]. This drug was investigated by Yamamura et al. because cholesterol depletion had an effect on autophagy. These authors showed that ezetimibe activates autophagy only in hepatocytes and intestinal epithelial cells, consistent with the presumed mechanism of action of the drug which involves the inhibition of cholesterol efflux Niemann-Pick-type C1-like 1 (NPC1L1) [29]. The studies suggest that, by inhibiting NPC1L1, ezetimibe reduces the recruitment of mTOR to the lysosome and therein inhibits mTORC1 activity to activate autophagy. Most exciting, the authors found that ezetimibe reduces cellular accumulation of ATZ in primary cultures of human hepatocytes engineered for expression of ATZ [28].

Studies by Shoji-Kawata et al. have identified a potent autophagy-inducing peptide which could potentially be utilized for drug development. Building on prior observations that the HIV protein Nef inhibits autophagy by directly interacting with the autophagy regulatory factor beclin 1 [30, 31], these authors succeeded in identifying an 18-amino acid Nef-interacting domain of beclin 1 and linked it to the Tat sequence to increase cell uptake. They went on to show that Tat-beclin 1 peptide is a potent inducer of autophagy and enhances the degradation of mutant huntingtin and several invasive bacterial and viral pathogens. Their findings suggest that Tat-beclin 1 may potentially be considered for diseases like ATD, other diseases caused by aggregation-prone proteins, and also possibly for certain infectious diseases.

A recent study showed that activation of autophagy using gene transfer of the transcription factor TFEB can ameliorate the proteotoxicity of ATZ. TFEB is a master gene that regulates autophagy and lysosomal gene expression (Figure 2). It appears to interact with mTORC1 on the lysosomal membrane and is negatively regulated by mTORC1 [32]. It was recently reported that TFEB induced autophagy-dependent ATZ clearance in a mammalian cellular model of ATD; using adenovirus-mediated gene transfer of TFEB in the PiZ mouse model, this strategy significantly promoted autophagic degradation of ATZ and reduced liver fibrosis* in vivo* [33]. These results provide further validation for the therapeutic strategy of enhancing autophagy for the liver disease caused by ATD and suggest that gene therapy of this type may also be possible to treat ATD liver disease in the future.

## 5. Potential Role for Autophagy-Enhancing Strategies in Other Genetic Liver Diseases

ATD serves as a paradigm for gain-of-toxic function liver disease resulting from the pathologic intracellular accumulation of misfolded proteins. Similar phenomena occur in two other rare liver diseases—fibrinogen storage disease and cholesterol ester storage disease. The defects in each of these conditions lead to abnormal intracellular accumulation of substrates that are hepatotoxic and can result in significant clinical liver disease. These disorders illustrate the essential role of autophagy in liver disease and provide insights into the potential efficacy of therapeutic strategies designed to enhance autophagic function.

*Fibrinogen Storage Disease*. Fibrinogen is a 340 kD dimeric plasma protein produced by hepatocytes that is a central component of the coagulation cascade. Each half of the dimer is composed of three polypeptide chains (termed A*α*, B*β*, and *γ*) that are assembled in the ER. Several mutations in the fibrinogen gamma chain have been reported (fibrinogen Brescia, Aguadilla, AI DuPont, and Angers) that are associated with fibrinogen storage disease, an autosomal-dominant disorder that is characterized by low circulating plasma fibrinogen levels and abnormal accumulation of fibrinogen components within the hepatocyte ER [34, 35]. Reminiscent of ATD-associated liver disease, patients with fibrinogen storage disease display characteristics of both loss of function (hypofibrinogenemia) and gain-of-toxic function (accumulation of fibrinogen components in ER). In addition to hematologic morbidity such as bleeding and thrombosis, patients may develop chronic liver injury that progresses to cirrhosis. Histologic examination of liver biopsy specimens reveals rounded, eosinophilic inclusions within hepatocytes that are weakly positive for phosphotungstic acid hematoxylin but are PAS-negative when pretreated with diastase. At the electron microscopic level these inclusions have a crystalline lattice organization and are now known to be composed of partially assembled fibrinogen molecules [34].

Experimental studies in yeast models of Aguadilla-variant fibrinogen storage disease by Kruse et al. demonstrated that disposal of mutant fibrinogen from the ER is mediated by two proteostasis mechanisms: endoplasmic reticulum-associated degradation (ERAD) and autophagy [36]. When the mutant *γ*D chain was expressed in yeast, initial clearance of the protein from the ER was performed by ERAD. However, once ERAD was saturated, *γ*D aggregates accumulated within the ER, and clearance of these aggregates was dependent on autophagy. These results suggest that the mutant fibrinogen Aguadilla behaves similarly to mutant ATZ in the fact that it accumulates within the secretory pathway, has a tendency to polymerize/aggregate, and is degraded by proteasomal and autophagic mechanisms. Based on these findings as well as work by the Perlmutter group that demonstrated amelioration of experimental ATD-associated liver disease by the autophagy-enhancing drug carbamazepine, Puls et al. recently conducted a small study of two patients with fibrinogen storage disease in which they found that administration of carbamazepine at antiepileptic doses was associated with normalization of alanine-aminotransferase and reduction of markers of apoptosis and necrosis [35].

*Cholesterol Ester Storage Disease.* Cholesterol ester storage disease (CESD) is an autosomal recessive condition that results from mutations in the* LIPA* gene, which encodes lysosomal acid lipase (LAL). LAL is a critical component of the low-density lipoprotein (LDL) receptor pathway that is responsible for hydrolyzing cholesterol esters that have been taken up into lysosomes.* LIPA* mutations associated with deficient LAL activity lead to intracellular accumulation of cholesterol esters and triglycerides [37]. The most severe form of CESD, known as Wolman disease, results from* LIPA* mutations that cause nearly complete absence of LAL activity and presents during infancy with hepatosplenomegaly, cholestasis, intestinal malabsorption, adrenal calcifications, and diffuse xanthomatosis [37, 38]. Less severe mutations result in a spectrum of disease collectively referred to as CESD, with age of presentation ranging from childhood to late adulthood. Liver manifestations include hepatomegaly and elevated transaminases occurring in combination with elevated serum cholesterol and triglyceride levels, low high-density lipoprotein (HDL) levels, and nonspecific gastrointestinal symptoms. In late-onset disease, adults may present with evidence of chronic liver disease and micronodular cirrhosis along with atherosclerosis. Liver biopsies may exhibit birefringent cholesterol ester crystals that are pathognomonic for this condition, as well as microvesicular steatosis that can be confused with nonalcoholic fatty liver disease [38, 39].

Lysosomal function is critical for efficient autophagic degradation of substrates, and recent experimental studies have provided evidence for the key role of autophagy in lipid metabolism. In a* C. elegans* system, the lysosomal lipases* lipl-1* and* lipl-3* (worm homologues of human LAL) are important to the mobilization of cytosolic fat through a specific autophagic process termed as lipophagy. Larvae that were double mutants for* lipl-1* and* lipl-3* accumulated three times the cytosolic fat as wild-type larvae, an observation that is reminiscent of cholesterol ester accumulation seen in CESD [40]. Another series of studies that examined lipid droplet mobilization in macrophages demonstrated that cholesterol efflux from macrophage foam cells is mediated by lipophagy and this mechanism is dependent on LAL function. Lipid-loaded macrophages treated with the specific LAL inhibitor Lalistat 1 demonstrated increased intracellular cholesterol ester load. Furthermore, autophagy-deficient Atg5-knockout macrophages and Atg5-knockout mice exhibited a significantly decreased ability to clear ^3^H-labeled cholesterol [41].

Severe CESD disease may lead to the need for liver transplantation, underscoring the need for novel therapies that can ameliorate the natural course of this condition. Lipid-lowering therapies including statins and cholestyramine have not been shown to reverse liver disease. More recently, efforts have focused on enzyme replacement therapy using recombinant human LAL (sebelipase alfa), which in experimental mouse studies appeared to reduce hepatic cholesterol ester load and reduced the presence of foamy macrophages in the liver, spleen, and intestines of these mice. This agent is currently in clinical trials [38, 39]. Due to theoretical concerns over the ability of enzyme replacement therapy to fully access target tissues in lysosomal storage disorders, an alternative approach has targeted TFEB. Spampanato et al. recently demonstrated in cell and mouse models of Pompe disease (a lysosomal storage disorder characterized by pathologic accumulation of intracellular glycogen) that adenoviral overexpression of TFEB induced lysosomal exocytosis and ameliorated excessive glycogen burden. These effects were blunted in autophagy-deficient cells [42]. Given the lysosomal dysfunction inherent to CESD and Pompe disease, we wonder whether CESD might be a novel target for autophagy-enhancing therapies, particularly TFEB-mediated clearance of aggregated substrates via lysosomal exocytosis and aggregate expulsion. Indeed, because TFEB activates multiple genes within the autophagolysosomal system, it is possible that its overexpression could potentially overcome the compromised lysosomal function in patients with partial enzyme deficiencies.

## 6. Conclusions

Because autophagy mediates turnover of damaged organelles and degradation of denatured and misfolded proteins and also mediates disposal of stored lipids, it represents a critical cytoprotective mechanism in homeostasis and during stress states. It appears to be particularly important in diseases caused by misfolded proteins and this may be because it is specialized for degradation of insoluble and soluble proteins. Recent studies have shown that strategies which enhance autophagy can reverse the consequences of proteotoxicity in the liver. These strategies include drugs and viral-mediated gene transfer approaches using the transcriptional activator of the autophagolysosomal system, TFEB. Because some of the drugs are FDA-approved and have been extensively used in clinical medicine, we should see results from clinical trials in the next several years.
